# Life of Harvey

**Published:** 1846-12

**Authors:** 


*Life of Harvey.*—William Harvey was descended from a respectable family in Kent. He was born at Folkstone on the 1st of April, 1578, the eldest of seven sons. He received his early education at Canterbury, and thence proceeded, when sixteen years old, to Caius College, Cambridge. After six years of study in the University, he went to Padua, and received there the degree of Doctor of Medicine in 1602.

On his return to England he was admitted in 1604 a Candidate, and in 1707 a Fellow, of the Royal College of Physicians. In 1609 he became physician to Saint Bartholomew's Hospital. In 1615 he was appointed to the Lumley and Cardwall Lectureship on Anatomy and Surgery, at the College of Physicians; and in the following year, in the Lectures which he read in this office, he first publicly explained his discovery and doctrine of the circulation of the blood. The account of his researches was first printed at Frankfort, with the title of "Exercitatio Anatomica de Motu Cordis et Sanguinis in animalibus," in 1628.

In 1623, Harvey, who had been for some time Physician Extraordinary to James I., was appointed to share the responsibilities of the Physician in Ordinary, and was promised the succession, on the first vacancy, to the dignity also.

In 1629, Charles I. sent him to travel with the Duke of Lenox; and on his return, in 1631, he became Physician to the King and royal household.

It is probable that, after this appointment, the greater part of Harvey's time was spent with the Court. The King encouraged his researches, and himself, with the nobles of his household, used to engage Harvey in the demonstration of his great discovery. It was also by the King's command that, in 1635, he dissected and recorded the examination of the body of Thomas Parr, who died at the age of 153 years.

When the civil war broke out, Harvey accompanied the King from London: he was present at the battle of Edgehill 1642, and thence went to Oxford, where he was shortly after incorporated a Doctor of Physic, and, in 1645, was made Warden of Merton College.

Within a few months, however, Oxford being surrendered to the Parliamentary troops, the former Warden was restored; and Harvey returned to London. He lived for some time with his brother Sir Eliab, who resided in the Poultry: and then passed many years in retirement at Lambeth and Richmond.

In 1651, with the same diffidence which had led him to delay the printing of his work upon the circulation of the blood for twelve years after he had completed the demonstration of the doctrine, he allowed his friend and biographer, Sir George Ent, to publish his "Exercitationes de Generatione Animalium." In 1652 he undertook to build and store, at his own expense, in the garden of the College of Physicians, a Museum and Library; which, being completed in the following year, he presented to the College, after a splendid banquet, to which he had invited all the fellows.

In 1654 Harvey was elected President of the College, but declined the honour on account of his age and infirmities; he still, however, attended the College meetings, and in 1656 gave to the College an estate of £56 a year, which had been left him by his father. In 1657, on the third of June, having entered his 80th year, he died: and the fellows of the College, who had already, in 1652, placed his statue in their Hall, accompanied his body far beyond the city walls on the way to its grave at Hempstead, in Essex.—*From Mr. Paget's Records of Harvey.*

